# Densely methylated DNA traps Methyl-CpG–binding domain protein 2 but permits free diffusion by Methyl-CpG–binding domain protein 3

**DOI:** 10.1016/j.jbc.2022.102428

**Published:** 2022-08-28

**Authors:** Gage O. Leighton, Elizabeth Marie Irvin, Parminder Kaur, Ming Liu, Changjiang You, Dhruv Bhattaram, Jacob Piehler, Robert Riehn, Hong Wang, Hai Pan, David C. Williams

**Affiliations:** 1 Department of Pathology and Laboratory Medicine, University of North Carolina at Chapel Hill School of Medicine, Chapel Hill, North Carolina, USA; 2 Toxicology Program, North Carolina State University, Raleigh, North Carolina, USA; 3 Department of Physics, North Carolina State University, Raleigh, North Carolina, USA; 4 Center for Human Health and the Environment, North Carolina State University, Raleigh, North Carolina, USA; 5 Department of Biology and Center for Cellular Nanoanalytics (CellNanOs), Universität Osnabrück, Osnabrück, Germany; 6 Department of Biomedical Engineering, Georgia Institute of Technology & Emory University of Medicine, Atlanta, Georgia, USA

**Keywords:** DNA methylation, epigenetics, DNA binding protein, gene regulation, single-molecule biophysics, atomic force microscopy (AFM), AFM, atomic force microscopy, bps, base pairs, CpG, cytosine-guanosine dinucleotide, IDR, intrinsically disordered region, MBD, methyl-CpG–binding domain, mCpG, methylated CpG, MSD, mean square displacement, NuRD, nucleosome remodeling and deacetylase complex, SAv-QD, streptavidin-coated quantum dot

## Abstract

The methyl-CpG–binding domain 2 and 3 proteins (MBD2 and MBD3) provide structural and DNA-binding function for the Nucleosome Remodeling and Deacetylase (NuRD) complex. The two proteins form distinct NuRD complexes and show different binding affinity and selectivity for methylated DNA. Previous studies have shown that MBD2 binds with high affinity and selectivity for a single methylated CpG dinucleotide while MBD3 does not. However, the NuRD complex functions in regions of the genome that contain many CpG dinucleotides (CpG islands). Therefore, in this work, we investigate the binding and diffusion of MBD2 and MBD3 on more biologically relevant DNA templates that contain a large CpG island or limited CpG sites. Using a combination of single-molecule and biophysical analyses, we show that both MBD2 and MBD3 diffuse freely and rapidly across unmethylated CpG-rich DNA. In contrast, we found methylation of large CpG islands traps MBD2 leading to stable and apparently static binding on the CpG island while MBD3 continues to diffuse freely. In addition, we demonstrate both proteins bend DNA, which is augmented by methylation. Together, these studies support a model in which MBD2-NuRD strongly localizes to and compacts methylated CpG islands while MBD3-NuRD can freely mobilize nucleosomes independent of methylation status.

The methyl-CpG–binding domain (MBD) family of proteins binds methylated DNA through a conserved domain that recognizes the symmetrically related methylcytosines in a cytosine-guanosine dinucleotide (CpG) (1). The structure of this domain bound to a single methylated CpG (mCpG) site has been determined for most members of the MBD family (2, 3, 4, 5, 6, 7, 8, 9). However, biologically relevant differential DNA methylation occurs within regions of the genome that contain tens to hundreds of CpG sites (CpG islands) (10, 11, 12, 13, 14). Furthermore, methylation of CpG islands in promoters and enhancers correlates with nucleosome occupancy, chromatin compaction, and associated gene silencing. Hence, we have investigated how MBD proteins bind and diffuse along these CpG islands to better understand the functional consequences in a more biologically relevant context.

In the current work, we focus on the structure and dynamics of the MBD2 and MBD3 proteins. These two highly homologous proteins arose from a duplication of the ancestral MBD present across the animal kingdom (1, 15). They contribute to the structure and function of the Nucleosome Remodeling and Deacetylase (NuRD) (16) complex that can reposition nucleosomes, deacetylate histones, and modify gene expression. The NuRD complex (17, 18, 19, 20) consists of a least six additional proteins, each of which has multiple paralogs that provide histone deacetylase activity (HDAC1/2), histone binding, and chromatin remodeling function (CHD3/4), and protein–protein interactions (GATAD2A/B, RBBP4/7, MTA1/2/3, CDK2AP1). The MBD2 and MBD3 proteins form distinct NuRD complexes that appear to have unique functional roles (16, 21, 22).

The two proteins show different levels of selectivity for mCpGs attributable primarily to a single amino acid change from tyrosine (MBD2) to phenylalanine (MBD3) within the DNA-binding site (Fig. 1) (1, 6, 23, 24). MBD2 shows up to 100-fold selectivity for a fully mCpG dinucleotide compared to an unmethylated CpG (1, 4, 25, 26). In contrast, MBD3 binds DNA with an overall much lower affinity and shows no or slight (3–5 fold) selectivity for an mCpG (6, 26). Consistent with this binding difference, previous genomic localization studies found that MBD2-NuRD predominantly binds at heavily mCpG islands associated with silenced genes, whereas MBD3 localizes to methylated and unmethylated CpG islands associated with expressed genes (23, 27, 28, 29). However, recent data suggests the alternative interpretation that both MBD2 and MBD3 depend on methylation for proper localization across the genome (30). Therefore, how methylation selectivity of the MBD2 and MBD3 proteins impacts this localization and function remains an open question in the field.

**Figure 1:**
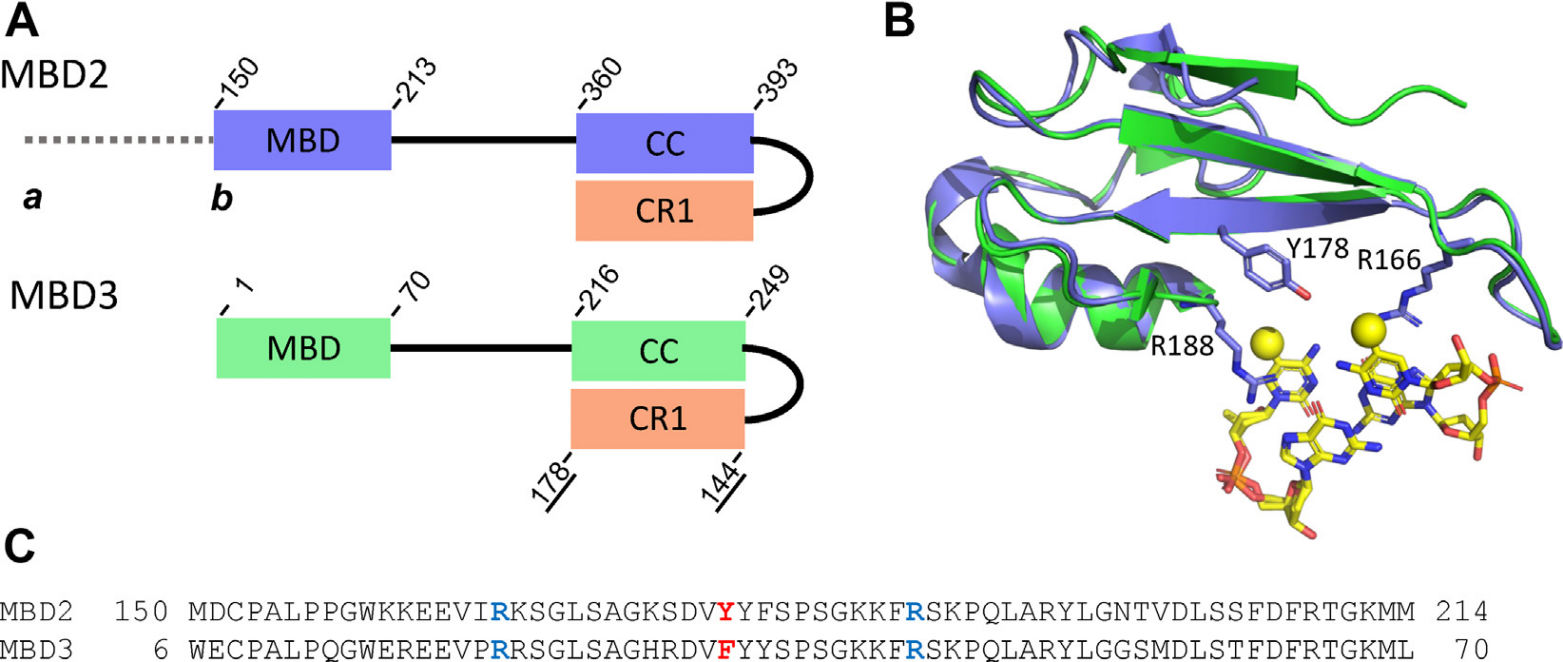
**MBD2 and MBD3 DNA-binding domains.***A*, cartoon representation of the MBD2 (*blue*) and MBD3 (*green*) protein constructs, which include the MBD and coiled-coil domains (CC) and a fusion to the coiled-coil domain from its native-binding partner GATAD2A (CR1, *orange*). We used the MBD2b isoform, which lacks a glycine-arginine–rich N-terminal region (*dashed line*) unique to the MBD2a isoform in mammals. *B*, a cartoon diagram depicts an alignment of the methyl-CpG–binding domains from MBD2 (*blue*) (8) and MBD3 (*green*) (9) bound to DNA (*yellow sticks*). The methyl carbons in the two methyl-cytosine bases of the CpG dinucleotide are shown as *yellow spheres*. The two arginine (R166 and R188) and 1 tyrosine (Y178) amino acids in MBD2 critical for selectively binding methylated DNA are shown in *sticks*. *C*, a sequence alignment of the two DNA-binding domains shows that the two arginine residues are conserved (highlighted in *blue*), but a phenylalanine replaces tyrosine in MBD3 (highlighted in *red*). MBD, methyl-CpG–binding domain; CpG, cytosine-guanosine dinucleotide.

In previous work, we used single-molecule analyses to study the behavior of the isolated MBD from MBD2 on various DNA substrates (31). Consistent with NMR and bulk biochemical studies, we found a remarkable difference in DNA bending and sliding of the MBD from MBD2 (MBD2_MBD_) on methylated and unmethylated DNA containing CpG islands. MBD2_MBD_ is mostly restricted to the mCpG islands, while it freely diffuses across unmethylated CpG islands. Furthermore, we also uncovered a novel role for the intrinsically disordered region (IDR) of MBD2 in DNA bending (25). The DNA-bending angle induced by both the MBD and a small portion of the IDR (MBD2_MBD+IDR_) on unmethylated CpG-rich DNA is larger than observed for CpG-free and further increases upon binding mCpG-rich DNA.

In separate structural studies of MBD3, we found that the MBD from MBD3 shows only weak selectivity for a single mCpG within a small (17 bp) dsDNA fragment (6). Based on a combination of chemical shift analyses, mutagenesis, and residual dipolar coupling measurements, we showed that MBD3 exchanges rapidly between CpG-specific and nonspecific binding modes, leading to chemical shift averaging between these two states. Hence, MBD3 recognizes an mCpG site, as evidenced by significant chemical shift changes, but does not strongly localize to this site when bound to DNA. This difference between the DNA-binding dynamics of MBD2 and MBD3 correlates with the prior localization studies that show both MBD2 and MBD3 localize to unmethylated CpG islands, while MBD2 more exclusively localizes to mCpG islands (23, 27, 28, 29).

Despite these recent studies, we do not know how the remaining, largely unstructured regions of MBD2 and MBD3 influence diffusion along DNA. Furthermore, it is unclear how reduced selectivity and binding affinity of MBD3 modifies its distribution and sliding on methylated and unmethylated CpG islands, which contain many CpG sites. To address these questions, in the current studies, we use a combination of biophysical techniques, including atomic force microscopy (AFM) imaging (32, 33, 34), DNA tightrope assays (35, 36, 37), and NMR (5, 6) to measure the binding and sliding of MBD2 and MBD3 on methylated and unmethylated DNA substrates.

## Results

### MBD2sc carries out unbiased 1D diffusion on CpG-free–rich DNA and subdiffusion on CpG-free DNA

**Figure 2:**
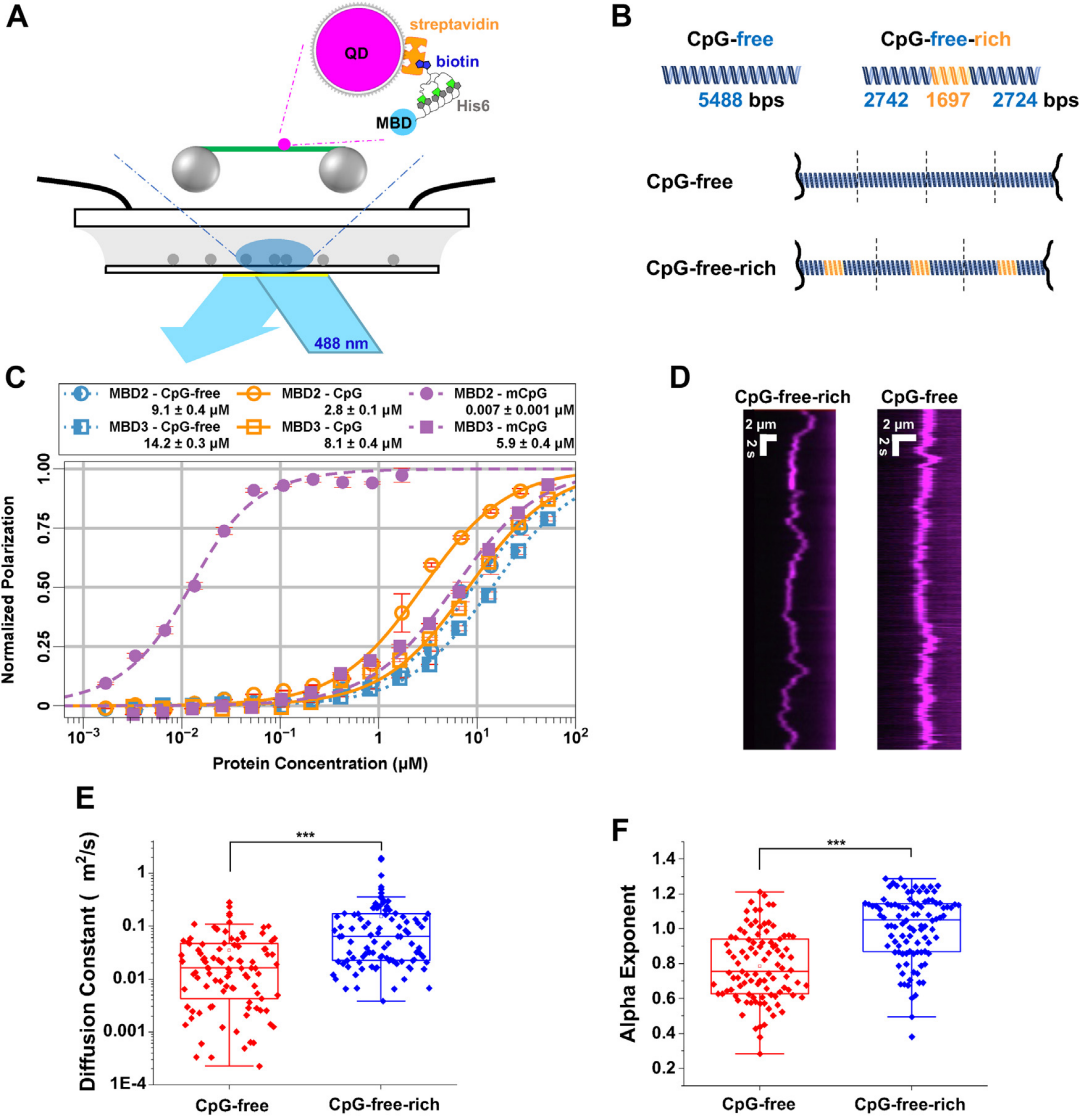
**Binding and diffusion of MBD2sc on unmethylated DNA substrates**. *A*, a schematic drawing of the DNA tightrope assay shows the flow-cell with an expanded representation of *red* (655 nm) QD-conjugated MBD2sc. ^BT^tris-NTA links the His_6_-tag on the MBD2sc proteins to streptavidin-coated–QD. *B*, a cartoon drawing depicts ligated DNA substrates for the DNA tightrope assay: CpG-free and CpG-free–rich. *C*, the binding affinity of MBD2sc or MBD3sc for unmethylated or methylated DNA as measured by fluorescence polarization. *D*, representative kymographs of QD-labeled MBD2sc on CpG-free and CpG-free–rich tightropes. Diffusion coefficients (*E*) and alpha exponents (*F*) of MBD2sc on CpG-free and CpG-free–rich DNA tightropes. ∗∗∗: *p* < 0.001. See Table 1 for detailed analysis. MBD, methyl-CpG–binding domain; CpG, cytosine-guanosine dinucleotide.

In the DNA tightrope assay, DNA molecules are stretched under hydrodynamic flow inside a flow cell. Anchoring of stretched DNA between poly-L-lysine–coated silica microspheres leads to the formation of DNA tightropes at an elongation of ∼90% of the DNA contour length (Fig. 2*A*). The spatial resolution of the DNA tightrope assay was estimated to be 16 nm (37). Uniquely, DNA tightropes created using tandemly ligated DNA allow us to directly correlate DNA-binding events with the underlying specific DNA sequences or structures such as three-stranded R-loops (31, 32, 37, 38). To study MBD proteins diffusion, we ligated linear DNA fragments to form DNA tightropes with CpG free or alternating CpG-free and CpG-rich regions (Fig. 2*B* and S8*A*). The results from our previous study revealed that the isolated MBD from MBD2, with or without a small portion of the adjacent IDR (MBD2_MBD_ and MBD2_MBD+IDR_), carry out unbiased 1D diffusion on CpG-rich DNA but undergoes subdiffusion on CpG-free DNA. In contrast, both proteins stably and statically bind to mCpG regions.

In this study, we purified a construct that contains almost the entire length of the MBD2b isoform plus the coiled-coil region from GATAD2A (MBD2sc) to investigate how these additional regions impact DNA binding and diffusion (Experimental Procedures, Fig. 1*A*). We previously found that the MBD2sc binds DNA with an approximately 100× higher affinity than MBD2_MBD_ (25). Consistent with these results, fluorescence anisotropy experiments showed that MBD2sc binds to DNA containing unmethylated, methylated, or no CpG site with equilibrium dissociation constants of 2.8 (±0.1 μM), 0.007 (±0.002 μM), or 9.1 (±0.4 μM), respectively (Fig. 2*C*). Hence, we questioned whether the additional binding affinity provided by the IDR in MBD2 would modify DNA binding and sliding. We directly addressed this question using the DNA tightrope assay. For the DNA tightrope assay, we first generated DNA substrates by tandemly ligating linear DNA fragments containing CpG-free or unmethylated CpG-free–rich sequences (Fig. 2*B*). Further, we conjugated His-tagged MBD2sc to streptavidin-coated quantum dots (SAv-QDs) through the multivalent chelator tris-nitrilotriacetic acid linker (Fig. 2*A*) (39). Following the formation of the DNA tightropes, we introduced QD-labeled MBD2sc into the flow cell. Analysis of MBD2sc on DNA tightropes revealed two populations (Table 1): apparently immobile throughout data acquisition and mobile molecules (Fig. 2*D*). To exclude that this apparently immobile population reflects aggregation, we categorized particles as a single protein or cluster based on their individual QD blinking rate (Table S1) which shows that only a small fraction comprises multiple proteins. To obtain diffusion coefficients for mobile MBD2sc on DNA tightropes, we tracked the position of MBD2sc-QDs on DNA by Gaussian fitting to kymographs (particle position *versus* time plots) (36, 40). We obtained diffusion coefficients and alpha exponents by fitting the mean square displacement (MSD) *versus* time. An alpha exponent of 1 indicates an unbiased random walk, and a value less than 1 indicates subdiffusion (41). The diffusion coefficients displayed by MBD2sc on the CpG-free DNA tightrope were significantly slower than those on DNA tightropes containing CpG sites (CpG-free–rich) (Fig. 2*E*). In addition, on the CpG-free–rich tightropes, MBD2sc displayed alpha exponents close to 1 (1.0 ± 0.2), indicating largely unbiased 1D diffusion on DNA (Fig. 2*F*, Table 2). As compared to CpG-free–rich DNA, the alpha exponents for MBD2sc on CpG-free DNA tightropes were slightly (*p* < 0.001) reduced (0.8 ± 0.2, Fig. 2*F*, Table 2). Overall, MBD2sc displayed slightly different diffusion ranges on CpG-free DNA tightropes compared to CpG-free–rich DNA tightropes containing multiple CpG sites (Fig. S3). In summary, MBD2sc shows more rapid and extensive 1D diffusion on CpG-free–rich DNA than CpG-free sequences.

**Table 1:** Fraction of statically bound MBD2sc and MBD3sc on unmethylated- and methylated-DNA tightropes

DNA	MBD2sc		MBD3sc	
	Static binding (%)	N	Static binding (%)	N
CpG-free–rich	23 ± 6	147	47 ± 7	123
CpG-free	19 ± 6	333	54 ± 8	240
mCpG-free–rich	96 ± 3	246	52 ± 10	345
mCpG-mini	90 ± 1	495	50 ± 8	158

The values represent mean ± SD from 2 to 3 experiments for each data set.

**Table 2:** Diffusion coefficient of MBD2sc and MBD3sc on different DNA substrates

DNA	MBD2sc			MBD3sc		
	D (μm^2^/s)	α exponent	N	D (μm^2^/s)	α exponent	N
CpG-free	0.04 ± 0.02	0.8 ± 0.2	99	0.04 ± 0.03	0.8 ± 0.3	199
CpG-free–rich	0.15 ± 0.05	1.0 ± 0.2	100	0.04 ± 0.01	0.9 ± 0.2	144
mCpG-free–rich	NA.	NA.		0.09 ± 0.02	1.0 ± 0.2	174
mCpG-mini	NA.	NA.		0.05 ± 0.02	0.9 ± 0.1	70

The values represent mean ± SD from 2 to 4 experiments for each data set.

### MBD2sc statically binds to mCpG regions

**Figure 3:**
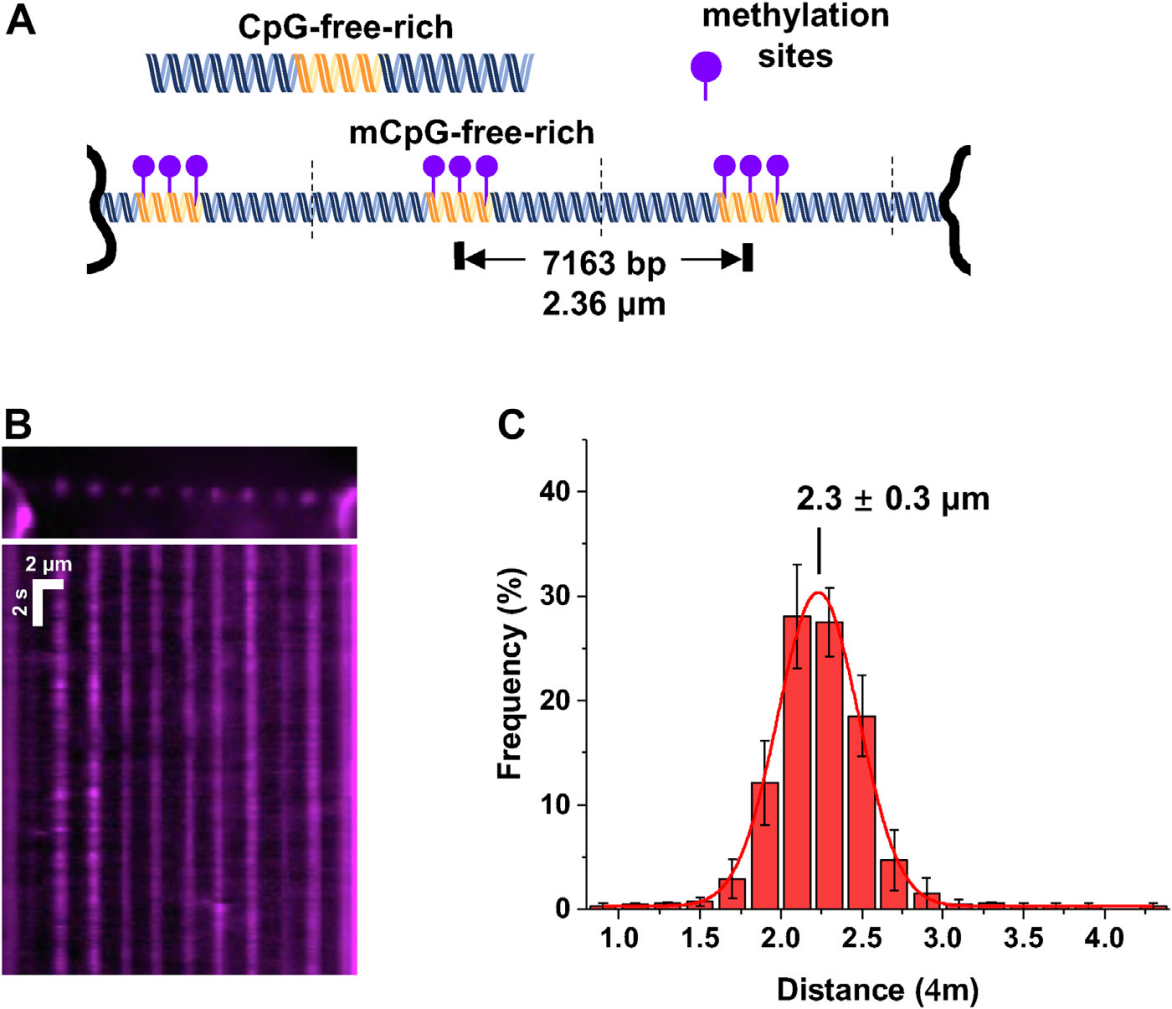
**MBD2sc becomes static at the methylated CpG-rich regions on DNA tightropes.***A*, a cartoon drawing of the CpG-free–rich DNA fragment (top panel) and ligated DNA substrate containing alternating mCpG-rich and CpG-free sequences (mCpG-free–rich, bottom panel) for the DNA tightrope assay. *B*, kymograph of MBD2sc on the ligated mCpG-free–rich DNA tightrope. *C*, Histogram of the distance between adjacent MBD2sc-QDs on mCpG-free–rich DNA tightropes. The *solid lines* represent Gaussian fit to the data (R^2^> 0.98) with a peak centered at 2.2 (±0.3 μm) (N = 461, the error bars represent mean ± SD). MBD, methyl-CpG–binding domain; CpG, cytosine-guanosine dinucleotide; mCpG, methylated CpG.

To evaluate how DNA methylation affects the dynamics of MBD2sc on DNA, we imaged QD-labeled MBD2sc on the CpG-free–rich DNA tightropes after methylation. Linear DNA substrates were methylated before ligation using CpG Methyltransferase (M.SssI) with SAM as a cofactor (Experimental procedures). We confirmed methylation of the linear CpG-free–rich DNA substrate by digestion with the methylation-sensitive HpaII restriction endonuclease (Fig. S1). We then tandemly ligated the mCpG-free–rich (mCpG-free-rich, Fig. 3*A*) and used it to form DNA tightropes between silica beads. Compared to unmethylated CpG-free–rich and CpG-free DNA, the binding density of MBD2sc increased approximately 4× on mCpG-free–rich DNA tightropes (Fig. 3*B*, S4*A*). This result is consistent with the preferential binding of MBD2sc to mCpG sites, as supported by binding affinity measurements (Fig. 2*C*). Notably, while the majority of MBD2sc observed on unmethylated CpG-free–rich DNA tightropes was mobile (77%), on mCpG-free–rich tightropes, the majority of the protein bound was apparently immobile (96%) throughout data acquisition (Fig. 3*B* and Table 1). Furthermore, the distance between adjacent proteins on the mCpG-free–rich DNA tightropes is Gaussian distributed, with the peak centered at 2.3 (±0.3 μm) (Fig. 3*C*). This spacing matches the calculated distances between adjacent mCpG-rich regions on DNA tightropes (Fig. 3*A*), considering that they are stretched to ∼90% of their contour length. Taken together, fluorescence imaging of MBD2sc on DNA tightropes establishes that MBD2sc recognizes mCpG islands through stable and apparently static binding.

### MBD2sc bends unmethylated and methylated DNA upon binding

**Figure 4:**
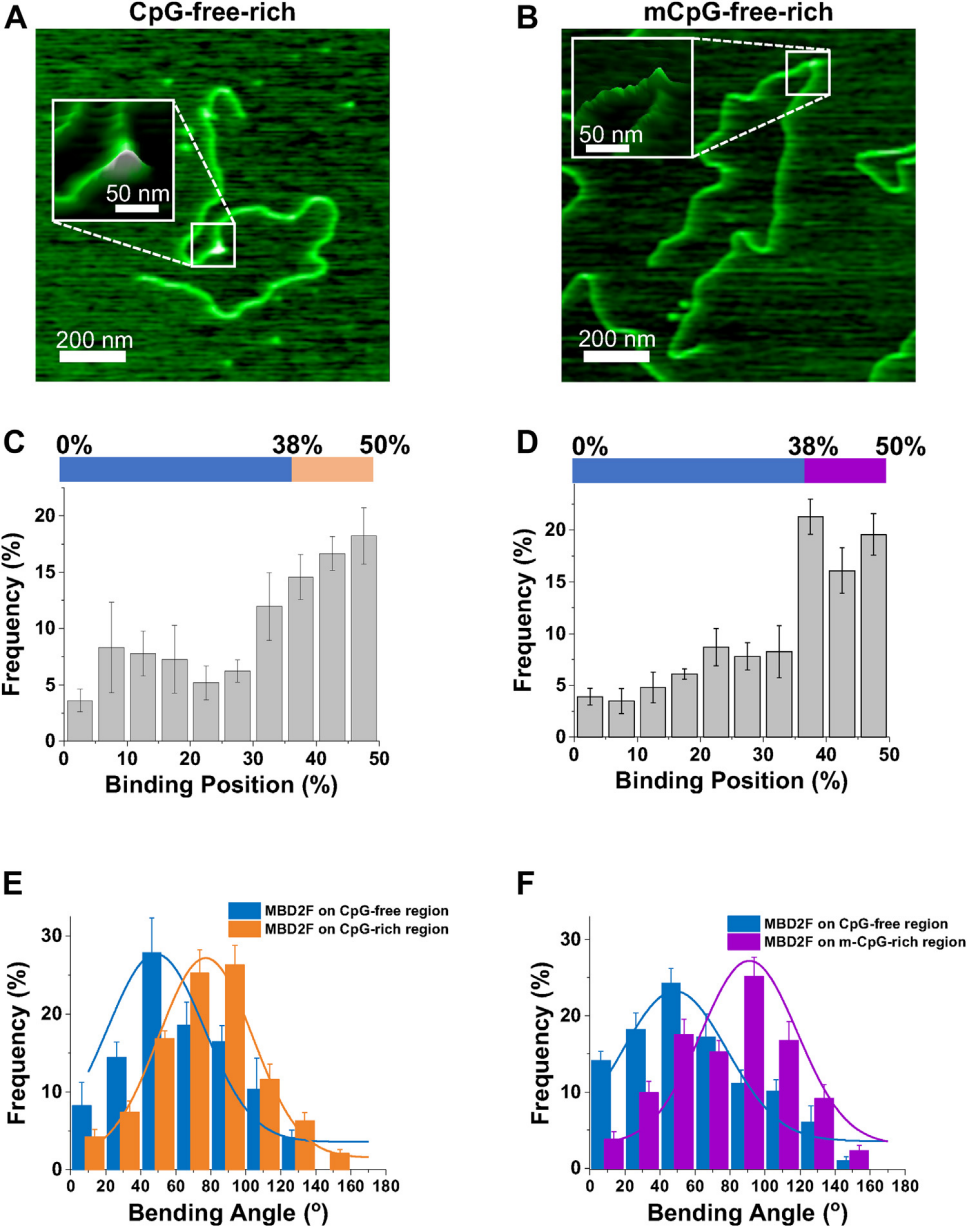
**MBD2sc induces DNA bending upon binding to CpG-free–rich and mCpG-free–rich DNA substrates.** Representative AFM images of MBD2sc on the linear CpG-free–rich (*A*) and on mCpG-free–rich DNA (*B*). The XY scale bar is 200 nm. Inset: an expanded 3-D image of the indicated region. *C* and *D*, *a*nalysis of the binding position of MDB2sc on the linear CpG-free–rich (*C*) and mCpG-free–rich (*D*) substrates. Over 49% of MBD2sc (N = 95 out of 192) binds to the CpG-rich region (38% to 50%) (*C*) and 60% of MBD2sc (N = 131 out of 230) binds to the mCpG-rich region (*D*). *E* and *F*, DNA-bending angles induced by MBD2sc upon binding to the CpG-free region (49° ± 28°, mean ± SD, N = 97) and CpG-rich region (77° ± 27°, N = 95) on the CpG-free–rich DNA and upon binding to the CpG-free region (49° ± 30°, N = 99) and m-CpG–rich region (91° ± 27°, N = 131) on the mCpG-free–rich DNA (*F*). MBD, methyl-CpG–binding domain; CpG, cytosine-guanosine dinucleotide; mCpG, methylated CpG.

To evaluate whether MBD2sc affects the DNA conformation upon binding, we applied AFM imaging in air to visualize the MBD2sc-DNA complexes on linear unmethylated or mCpG-free–rich DNA (Fig. 4). Based on the central location of the CpG-rich region, we identify proteins bound to this region when they are located between 38 to 50% from either end of the linearized DNA. The heights of MBD2sc on DNA (0.73 ± 0.05 nm) was significantly (*p* <0.001) greater than that of dsDNA itself (0.31 ± 0.03 nm), allowing us to unambiguously identify the protein–DNA complexes and determine whether they are located within the CpG-rich or CpG-free regions. The binding position analysis revealed that MBD2sc preferentially binds to the CpG-rich region on both the unmethylated and methylated DNA substrates (Fig. 4, *C* and *D*). Similar to what we discovered previously for the isolated MBD2_MBD_, MBD2sc induced DNA bending when localized to the CpG-free and CpG-rich regions (Fig. 4*E*). The DNA-bending angles caused by MBD2 at the CpG-rich region (77 ± 27°) were slightly larger than observed on the CpG-free region (49 ± 28°, Table 3). In contrast, MBD2sc induced significantly (*p* < 0.001) larger bending angles (91 ± 27°) at the mCpG-rich region (Fig. 4*F* and Table 3). In summary, MBD2sc bends CpG-free and unmethylated CpG-rich DNA substrates, promoting additional DNA bending when interacting with mCpG DNA.

**Table 3:** Summary of DNA-bending angles induced by MBD2sc and MBD3sc binding

DNA	MBD2sc		MBD3sc	
	Bending angle (°)	N	Bending angle (°)	N
Unmethylated				
CpG-free region	49 ± 28	97	47 ± 26	168
CpG-rich region	77 ± 27	95	68 ± 28	119
Methylated				
CpG-free region	49 ± 30	99	36 ± 26	168
CpG-rich region	91 ± 27	131	82 ± 30	138

The values represent mean ± SD from 2 to 3 experiments for each data set. The significance values regarding the difference among the datasets are reported in Supplementary Figure 7.

### MBD3 diffuses freely on mCpG DNA substrates

**Figure 5:**
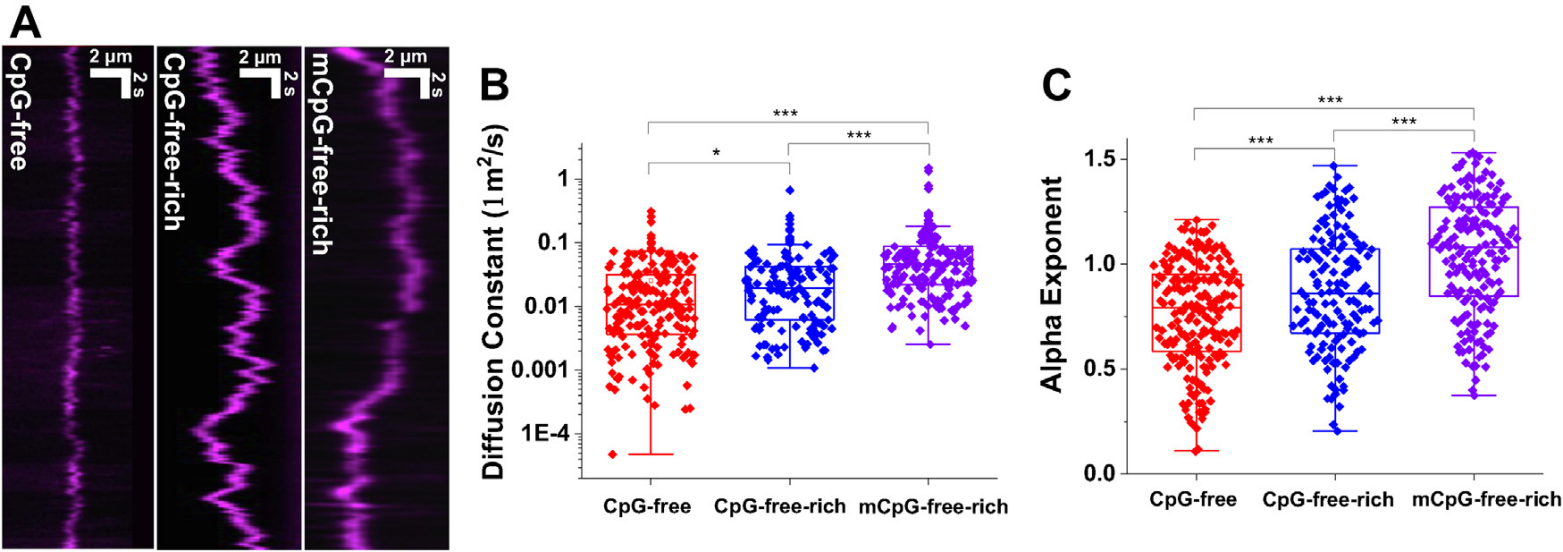
**DNA tightrope assay of MBD3sc on unmethylated and methylated DNA substrates.***A*, representative kymographs of MBD3sc on different DNA tightropes. *B* and *C*, diffusion coefficients (*B*) and alpha exponents (*C*) of MDB3sc on CpG-free, CpG-free–rich, and mCpG-free–rich DNA tightropes. ∗: *p* < 0.05; ∗∗∗: *p* < 0.001. See Table 1 for detailed analysis. MBD, methyl-CpG–binding domain; CpG, cytosine-guanosine dinucleotide; mCpG, methylated CpG.

Previous studies showed that MBD3 lacks a high affinity for CpG islands regardless of methylation status. Our recent study by NMR showed that MBD3 binds DNA with low affinity and shows only a slight preference for methylated DNA (6). Fluorescence anisotropy experiments demonstrated that MBD3sc binds to DNA containing unmethylated, methylated, or no CpG sites with equilibrium dissociation constants of 8.1 (±0.4 μM), 5.9 (±0.4 μM), and 14.2 (±0.3 μM), respectively (Fig. 2*C*). To investigate how MBD3 differs from MBD2 in binding to CpG islands, we analyzed its binding to DNA tightropes using the same set of DNA substrates (CpG-free, CpG-free–rich, and mCpG-free–rich, Fig. 5*A*). Similar to MBD2sc, MBD3sc showed both static and mobile populations on these DNA substrates (Table 1). Compared to MBD2sc, MBD3sc static binding rates appeared unaffected by CpG island presence (47 ± 7% on CpG-free–rich and 54 ± 8% on CpG-free) and methylation status (52 ± 10% on mCpG-free–rich) (Table 1). MBD3sc shows a much lower fraction of static binding events on mCpG-free–rich tightropes than MBD2sc.

We fit the MSD *versus* time to obtain the diffusion coefficients and alpha exponents of mobile MBD3sc on different DNA substrates. MBD3sc showed slower diffusion on CpG-free–rich and CpG-free DNA tightropes than MBD2sc (Fig. 5 and Table 2). Furthermore, the alpha exponents of MBD3sc on CpG-free–rich and CpG-free DNA were slightly less than 1, suggesting constrained and subdiffusive motion. Surprisingly, MBD3sc showed faster diffusion on mCpG-free–rich tightropes and an alpha exponent of 1.0 (±0.3). This faster diffusion of MBD3sc on mCpG-free–rich DNA contrasts with the apparently static binding by MBD2sc. Additionally, we measured the diffusion ranges of MBD3sc on these DNA tightropes (Fig. S3*B*). MBD3sc appears to diffuse over a more extensive range on mCpG-free–rich than on CpG-free and CpG-free–rich DNA substrates (*p* < 0.001). It is worth noting that the diffusion range of MBD3sc on the methylated DNA tightrope was increased to 1.6 (±0.8 μm). Taken together, MBD3sc appears to exhibit subdiffusion on unmethylated DNA substrates. Moreover, the majority of MBD3sc on mCpG-free–rich DNA is mobile and moves through unbiased 1D diffusion.

### Unmethylated and methylated DNA bending upon MBD3sc binding

**Figure 6:**
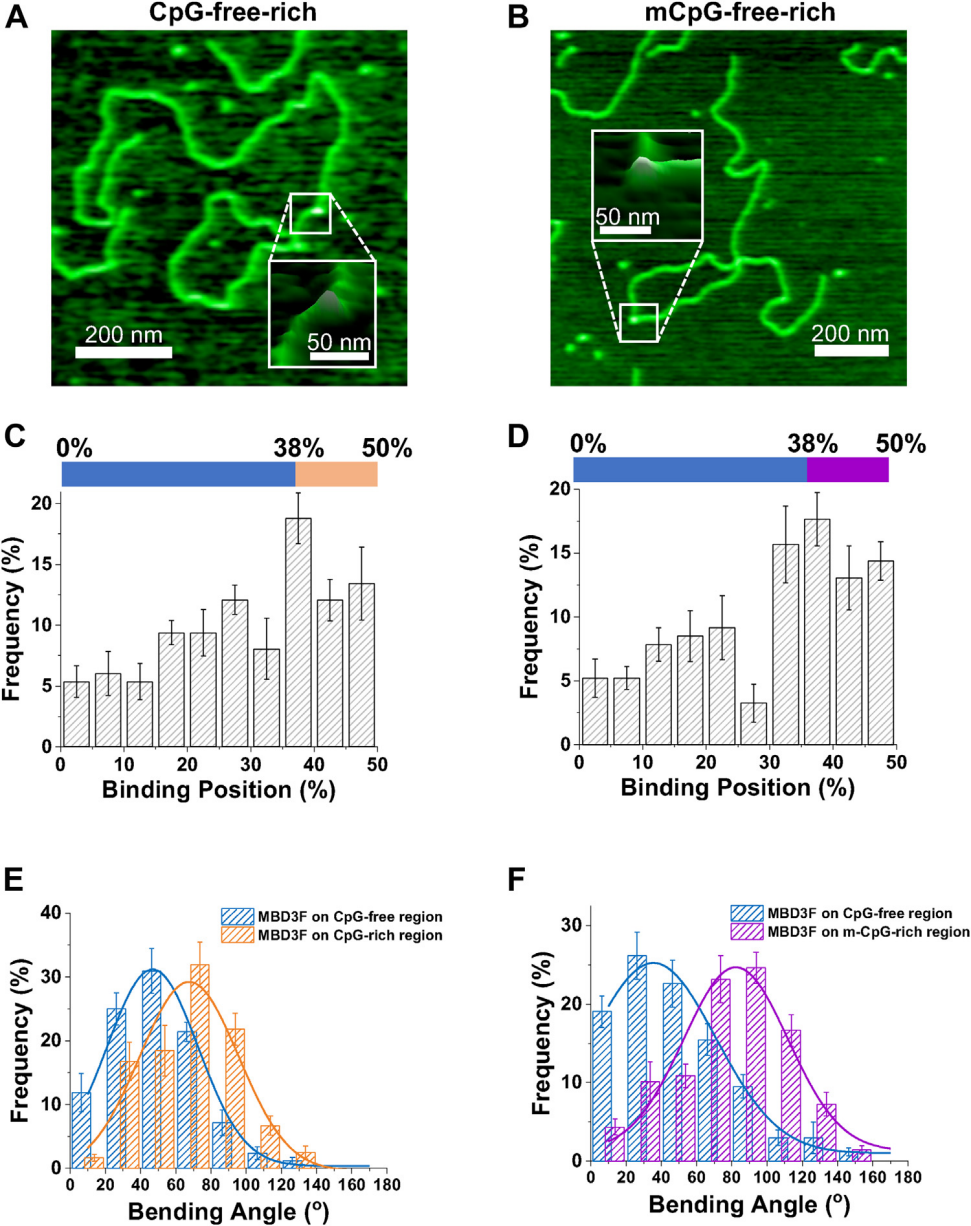
**MBD3sc induces DNA bending upon binding to CpG-free–rich and mCpG-free–rich DNA substrates.***A* and *B*, representative AFM images of MBD3sc on the linear CpG-free–rich (*A*) and on mCpG-free–rich DNA (*B*). The XY scale bar is 200 nm. Inset: An expanded 3-D image of the indicated region. *C* and *D*, analysis of the binding position of MDB3sc on the linear CpG-free–rich and mCpG-free–rich substrates. Over 41% of MBD3sc (N = 119 out of 287) binds to the CpG-rich region (38% to 50%) (*C*) and 45% of MBD3sc (N = 138 out of 306) binds to the mCpG-rich region (*D*). *E* and *F*, DNA-bending angles induced upon MBD3sc binding to the CpG-free region (47° ± 26°, mean ± SD, N = 168) and CpG-rich region (68° ± 28°, N = 119) on CpG-free–rich DNA (*E*) and upon binding to the CpG-free region (36° ± 26°, N = 168) and m-CpG–rich region on mCpG-free–rich DNA (82° ± 30°, N = 138) on the mCpG-free–rich DNA (*F*). MBD, methyl-CpG–binding domain; CpG, cytosine-guanosine dinucleotide; mCpG, methylated CpG.

To further investigate the DNA conformational changes upon MBD3sc binding, we used AFM to visualize MBD3sc–DNA complexes on different substrates (Fig. 6, *A* and *B*). The binding position analysis showed that MBD3sc displayed a slight preference for the CpG (42%, Fig. 6*C*) and mCpG (45%) DNA regions (38% to 50% of DNA length) (Fig. 6*D*) though it was weaker than MBD2sc. Similarly, the bending angles induced upon MBD3sc binding at the CpG-rich region (68 ± 28°) and mCpG-rich region (82 ± 30°) were significantly larger (*p* < 0.001) than on CpG-free region (47 ± 26° and 36 ± 26°) (Fig. 6, *E* and *F* and Fig. S5). Though the DNA-bending angle induced by MBD3sc at the mCpG-rich region was similar to MBD2sc, it is distinctly smaller at the CpG-free region than MBD2sc (Fig. S5). To sum up, MBD3sc bends CpG-free and CpG-free–rich DNA upon binding. Similar to MBD2sc, MBD3sc promotes additional bending when binding to the mCpG-rich region.

### MBD3sc forms DNA–DNA pairing tracts upon binding on methylated DNA substrates

**Figure 7:**
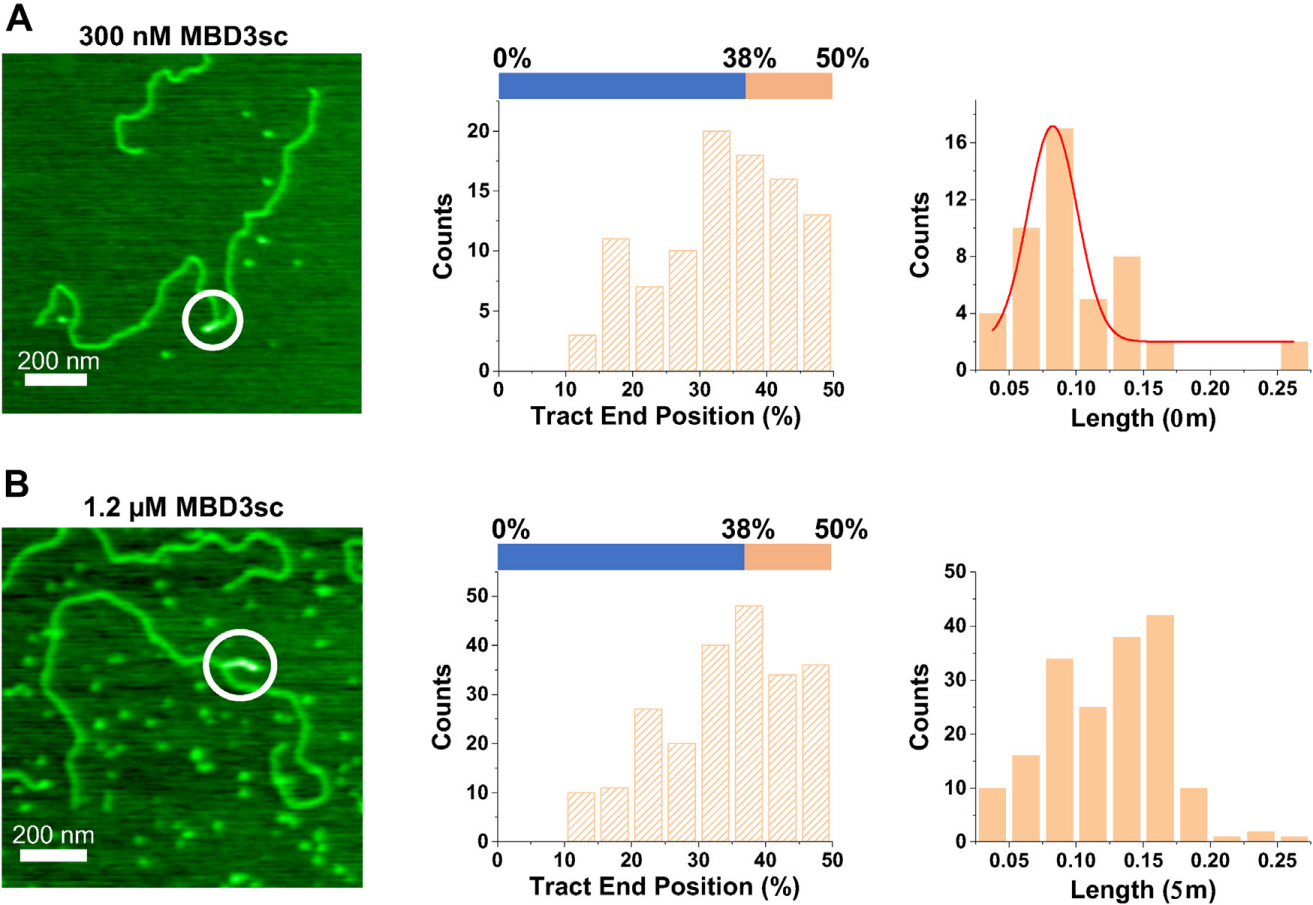
**DNA-DNA pairing tracts formed upon MBD3sc binding on the methylated CpG-free–rich DNA substrate.***A* and *B*, representative AFM images of a DNA-DNA pairing tract (*circle*, left panels), tract positions (middle panels), and tract lengths (right panels) upon MBD3sc binding on the linear mCpG-free–rich DNA at a lower (*A*) and higher (*B*) MBD3sc concentration. The XY scale bar is 200 nm. 48% (N = 48) and 52% (N = 226) of the tracts formed in the methylated CpG-rich region at the low and high MBD3sc concentration, respectively. The length of tracts formed upon MBD3sc binding on the linear mCpG-free–rich DNA is 0.08 ± 0.02 μm and 0.13 ± 0.04 μm (mean ± SD) at the low and high MBD3sc concentration, respectively. MBD, methyl-CpG–binding domain; CpG, cytosine-guanosine dinucleotide; mCpG, methylated CpG.

Although MBD3sc bends DNA similarly to MBD2sc, we observed DNA-DNA pairing tracts formed upon MBD3sc binding (8% of 1059 DNA molecules), but not MBD2sc (Fig. 7*A*). Approximately, half of the DNA–DNA pairing tracts mediated by MBD3sc (300 nM) were formed within the mCpG-rich region (48%, N = 48). The average tract length was 0.08 (±0.02 μm), which was much shorter than the length of the CpG-rich region (1697 base pairs (bps), ∼0.56 μm). To confirm that DNA–DNA pairing tract formation is due to MBD3sc binding, we increased the MBD3sc protein concentration (from 300 nM to 1.2 μM) but kept the DNA concentration (∼0.5 ng/μl) constant (Fig. 7*B*). As a result, the percentage of tract formation increased from 8% to 25% (N = 278 out of 1130 DNA molecules), and 52% of the DNA–DNA pairing tracts (N = 226, Fig. 7*B*) formed in the mCpG-rich region (38% to 50% from DNA end), with a significant increase of the tract length to 0.13 (±0.04 μm) (Fig. 7*B*). In comparison, MBD2sc formed large clusters, not individual DNA–DNA pairing tracts, at the same protein concentration (1.2 μM) and reaction conditions (Fig. S6). Hence, MBD3sc shows a unique capacity to induce DNA–DNA pairing tracts, the majority of which form within the mCpG-rich region and the length of these tracts expands with increasing protein concentration.

### MBD2 exchanges rapidly between neighboring mCpG sites

**Figure 8:**
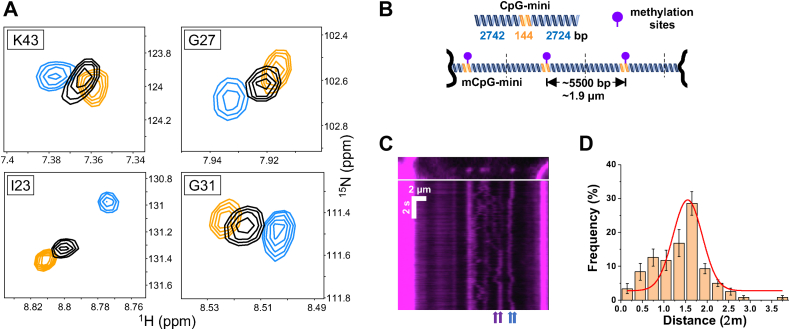
**MBD2sc exchanges between neighboring mCpG sites demonstrated by NMR and the DNA tightrope assay.***A*, overlay of ^1^H-^15^ N HSQC spectra of select amide resonances corresponding to the MBD2 DNA-binding domain (V22, I23, G27, K43) upon binding to DNA with either the first (*orange*), second (*blue*), or both (*black*) CpG sites methylated. With both sites methylated, we observe an averaging of the peak indicating rapid exchange on the order of ≤1 ms. *B*, schematic drawing of the ligated mCpG-mini DNA substrate used for the DNA tightrope assay. *C*, representative fluorescence image (top) and kymograph (bottom) of MBD2sc on the mCpG-mini DNA tightrope. *Arrows* at the bottom pointing to pausing events. *D*, histogram of the distance between transient pausing events on mCpG-mini DNA tightropes (N = 83). MBD, methyl-CpG–binding domain; CpG, cytosine-guanosine dinucleotide; mCpG, methylated CpG.

While the DNA tightrope assays demonstrate apparently static binding of MBD2sc to mCpG islands on the seconds to minutes timescale, in previous work, we found that the isolated MBD from MBD2 and MBD4 can exchange between two closely spaced mCpG sites on fast NMR timescales (sub-millisecond) (5). To determine whether MBD2sc likewise exchanges rapidly between two neighboring mCpGs, we collected NMR spectra of MBD2sc bound to DNA substrates with CpG sites separated by 14 bps (Fig. 8*A*, Experimental Procedures). This spacing is larger than but within 1 SD of the average spacing in the CpG-rich DNA (Fig. S2*B*). Additionally, this spacing place the mCpG sites on nearly opposite sides of B-form DNA.

As shown in Fig. 8*A* and S7, selective amide resonances corresponding to the MBD2 DNA-binding domain (V22, I23, G27, K43) in the 2D ^15^N-HSQC spectrum demonstrate distinct chemical shifts when bound to DNA with either 1 or the other of the CpG sites methylated. Importantly, these chemical shift differences allow us to interrogate the rate of exchange between the two sites. When both sites are methylated, these same amides show a single peak at a position that falls between those for the singly methylated DNA. Hence, MBD2sc shows chemical shift averaging consistent with rapid exchange between the two adjacent mCpG sites. This observation indicates the exchange rates are much faster than the difference in chemical shift between the two states (k >> δ_A_ – δ_B_), which is in the millisecond to sub-millisecond timescale. This rapid exchange contrasts with the apparent static binding for seconds to minutes as observed by single-molecule fluorescence on DNA tightropes. Therefore, we conclude that MBD2sc exchanges rapidly between neighboring CpGs, but the distance between these sites on the mCpG-rich substrate (10 bps, Fig. S2*B*) is below the spatial and temporal resolution of the fluorescence microscope. The high number of CpG sites within the island effectively trap MBD2 resulting in an apparently static binding behavior.

To further explore this hypothesis, we constructed a DNA tightrope substrate with far fewer CpG sites (CpG-mini, Fig. 8*B*) which would not be considered a CpG island as statistically defined (10, 42). Importantly, the relative spacing between CpG sites within the CpG-mini is comparable to that of the CpG-rich region (Fig. S2*B*). We observed slightly fewer apparently static binding events when MBD2sc bound to this mCpG-mini DNA tightrope (90%) compared to the mCpG-free–rich substrate (96%, Table 1). Additionally, MBD2sc showed transient pausing (59% percent mobile) and transition events (41% percent) between separate regions (Fig. 8*C*), which were not observed on the mCpG-free–rich DNA (Fig. 3*B*). The distance of MBD2sc between pausing events was measured at 1.6 (±0.5 μm) (Fig. 8*D*), which is within error of the estimated distance between CpG-mini segments (1.9 μm, Fig. 8*B*). Lastly, MBD3sc diffusion on the mCpG-mini DNA tightropes exhibited unbiased free 1D diffusion (Fig. S8) over a range comparable to the mCpG-free–rich tightropes. These observations demonstrate that the size of mCpG islands may impact the frequency of apparently static binding by MBD2sc and its ability to transition between CpG-rich regions. While MBD2sc can rapidly exchange between closely spaced mCpG sites, it cannot escape large mCpG islands. In contrast, MBD3sc diffuses rapidly across both small and large mCpG regions.

## Discussion

The MBD2 and MBD3 proteins provide structural and DNA binding functionality to the NuRD complex. The two proteins form mutually exclusive NuRD complexes, but only MBD2 shows strong selectivity for mCpG dinucleotides. The IDR and coiled-coil domains of each protein bridge between the histone deacetylase core and chromatin remodeling subcomplexes of NuRD. In these studies, we used protein constructs that incorporate the MBD N-terminal DNA-binding domain, the central IDR, and its C-terminal coiled-coil domain fused to its native binding partner, the coiled-coiled domain from GATAD2A (Fig. 1*A*). In previous work, we showed that the coiled-coil domains of MBD2 and MBD3 form stable heterodimeric complexes with the coiled-coil domain of GATAD2A (43, 44). In addition, we found that connecting the GATAD2A and MBD2 coiled-coil domains with a short linker generates a highly stable monomeric domain, such that these single-chain constructs (MBD2sc and MBD3sc) are more stable in solution. Notably, including the disordered and coiled-coil regions increases the binding affinity of MBD3 for methylated DNA from a K_D_ ∼ 50 mM (6) to ∼ 5.9 mM (Fig. 2*C*), which facilitates single-molecule studies under dilute conditions. Furthermore, the MBD2sc construct binds methylated DNA with approximately 100-fold greater affinity than the isolated MBD domain (25). These highly homologous constructs allow us to compare the binding and diffusion of MBD2 and MBD3 on different unmethylated and methylated DNA substrates, probing the relationship between DNA-binding behavior and the length and density of the CpG-rich region.

We find that MBD2sc statically binds to the mCpG-free–rich DNA tightropes over the 30-s timescale of these experiments with a physical spacing (2.2 ± 0.3 μm) consistent with the distance between adjacent mCpG-rich regions (2.36 μm). We further confirm that MBD2sc localizes to the mCpG-rich region by AFM (Fig. 4). These data show that MBD2sc preferentially and stably binds to the CpG-rich DNA with 2-fold higher occupancy than CpG-free regions. As expected, methylating the CpG-rich DNA leads to a further increase in occupancy on the CpG-rich region. In addition, localization to the mCpG-rich region corresponds with an increase in DNA bending (Fig. 4).

In contrast, MBD3sc undergoes unbiased 1D diffusion even when bound to mCpG-rich DNA. Moreover, the rate of diffusion of MBD3sc increases on methylated DNA (Fig. 5*B* and Table 2). Previous NMR studies demonstrated that MBD3 rapidly exchanges between CpG-specific and nonspecific binding modes (6). The current studies show that this CpG-specific binding mode promotes rapid diffusion along the CpG-island. Consistent with this hypothesis, both MBD2sc and MBD3sc show reduced and more restricted diffusion on CpG-free DNA (Figure 2, Figure 5). Hence, the interaction with unmethylated CpG-rich DNA permits free diffusion for both proteins, whereas methylation restricts diffusion by MBD2 while allowing free diffusion by MBD3.

Since NMR analysis shows that MBD2sc rapidly exchanges between neighboring mCpGs, we questioned if reducing the CpG island’s size or the number of CpGs would decrease the static binding on DNA tightropes. The 140 bps CpG-mini DNA substrate does not meet the minimal length of a statistically defined CpG island (42) when either fully methylated (11 mCpGs) or partially methylated (2 mCpGs). Nonetheless, MBD2sc shows mainly static binding on the methylated and partially mCpG-mini (Fig. 8). Interestingly, we noticed transitions between pausing events, indicating the diffusion of MBD2sc from 1 CpG-mini region to another across the CpG-free region (Fig. 8*C*). Therefore, short mCpG regions may not sufficiently trap the MBD2–NuRD complex for biological function.

Together, these data support a model for the localization and distinct functional roles of the two proteins. MBD2 strongly localizes to large and heavily mCpG islands, where it can recruit NuRD to drive nucleosome positioning, histone deacetylation, and ultimately chromatin compaction. Conversely, MBD3 remains mobile on methylated DNA, allowing NuRD to freely reposition nucleosomes across or perhaps even moving them away from mCpG islands. This model correlates with genomic localization studies of MBD2 and MBD3 which have shown that MBD2 strongly localizes to mCpG islands associated with silenced genes, while MBD3 localizes to unmethylated CpG islands associated with open chromatin and expressed genes (27, 28, 29). We propose that trapping of MBD2 in heavily mCpG islands drives MBD2-NuRD to preferentially move nucleosomes into these same regions. Importantly, MBD2 does not bind statically to a single mCpG, but instead continues to move nucleosomes within the methylated island, ultimately stabilizing a nucleosome-rich and compacted state. In contrast, MBD3 does not get trapped by CpG-rich regions, either methylated or unmethylated, such that MBD3-NuRD can move nucleosomes both into and out of these regions which does not drive a compacted state.

Furthermore, our model suggests that MBD3-NuRD opposes the function of MBD2-NuRD by freely mobilizing nucleosomes across methylated regions. This role of MBD3-NuRD may prevent aberrant silencing by MBD2-NuRD until a minimal threshold of methylation density across a large region has been reached. Hence, the MBD2–NuRD and MBD3–NuRD remodeling complexes have distinct functional roles that, at least in part, reflect the dynamics and distribution of MBD2 and MBD3 on methylated and unmethylated DNA.

We previously identified that MBD2 induced bending of various DNA substrates. In the studies here, we find that including the disordered and coiled-coil regions induce additional bending. We propose that the positively charged unstructured regions help neutralize the phosphate backbone, releasing water and ions which contributes to bending of the DNA (45, 46). Of note, structural studies of MBD2 and MBD3 bound to small DNA fragments have not shown evidence of DNA bending (4, 8, 9), suggesting that the bending we observe does not involve intercalation of the DNA by the protein and is more apparent over longer length-scales. In a chromatin, DNA exists as various prebent shapes such as DNA-wrapped nucleosomes or 3D loops. Therefore, preferential binding to bent DNA could promote or at least allow localization of MBD2-NuRD to nucleosome-rich and compacted regions of the genome. Furthermore, the small footprint of MBD on DNA (4) along with preferentially binding to bent DNA may allow MBD2 and MBD3 to bind DNA wrapped around nucleosomes. Structural (45, 46) and biophysical analyses of MBD2 bound to nucleosomes could help clarify these possibilities.

Tract formation by MBD3sc also suggests that at high concentrations, MBD3 may still promote chromatin compaction at mCpG islands. Furthermore, alternative interaction partners, such as associated transcription factors (47, 48, 49, 50, 51, 52, 53), could play a dominant role in localizing MBD3–NuRD complexes. Finally, these data confirm that a methylated region of only 11 CpGs is sufficient to cause MBD2sc to pause for a significant period (tens of seconds). Therefore, a few mCpGs, perhaps in conjunction with transcription factor recruitment, could be sufficient for gene silencing by the MBD2–NuRD complex.

Hence, we propose that the gene duplication event that generated MBD2 and MBD3 paralogs in vertebrates permitted the sub-specialization of these two related complexes. MBD2 demonstrates a much higher affinity for and likely more effectively compacts chromatin of mCpG islands. In contrast, MBD3 has lost methylation selectivity and likely more efficiently maintains open chromatin at unmethylated and transcriptionally active CpG islands. Therefore, MBD2-NuRD contributes to gene silencing at methylated promoters, while MBD3-NuRD helps maintain open chromatin at unmethylated, and possibly methylated, CpG island-associated promoters and enhancers.

## Experimental procedures

### Protein expression and purification

Human MBD2 (amino acids 150–393) and MBD3 (amino acids 1–249) were cloned into a modified pET32a vector with N-terminal thioredoxin, hexahistidine, and TEV protease sites. The constructs incorporated the coiled-coil domain of GATAD2A (amino acids 137–178) at the C-terminus to create a single-chain fusion, as described previously (25). After transforming the plasmids into Rosetta2 (DE3) *Escherichia coli*, the cells were grown at 37 °C in LB (unlabeled) or M9 minimal media (^2^H-, ^15^N-labeled) to an A_600_ ∼0.8 and induced with 1 mM IPTG for 2.5 or 4 h, respectively. Pelleted cells were either frozen at -20 °C or immediately lysed in 20 mM Tris pH 8.0, 1 M NaCl, and cOmplete Protease Inhibitor Cocktail (Roche) by sonication. Using standard procedures, we clarified the lysate by centrifugation at 16000×*g* for 30 min before purifying the protein by nickel affinity chromatography. For NMR studies, we digested the uniformly ^2^H- and ^15^N-labeled protein with TEV protease overnight at 4 °C and removed the thioredoxin fusion tag by a second nickel affinity chromatography step. We then purified both labeled and unlabeled samples by gel filtration (Superdex-75, GE Life Sciences) and verified the purity by SDS-PAGE.

### NMR spectroscopy

We purchased complementary 38 bps DNA oligonucleotides from Integrated DNA Technologies (IDT) with two CpG sites separated by 14 bps. Three double-stranded DNA samples were prepared with either the first, second, or both sites symmetrically methylated (GGAGGCGCT(mC)GGCGGCAGCCTGGAA(mC)GGAATTCTTCTA). The DNA was annealed and purified by anion exchange chromatography (MonoQ 10/100, GE Healthcare), with the concentration determined by UV before adding at 10% molar excess to ^2^H- and ^15^N-labeled MBD2sc. The protein:DNA complexes were buffer exchanged into 10 mM NaPO_4_, pH 6.5, 150 mM NaCl, 0.02% sodium azide, 1 mM DTT, and 10% ^2^H_2_O at a final protein concentration of 0.3 mM. ^15^N-HSQC spectra were collected on a Bruker Avance III 850 MHz magnet at 25 °C, processed with NMRPipe, and analyzed in CcpNMR (54, 55).

### Fluorescence anisotropy

Protein binding to a 17-bp 6-FAM-labeled DNA (IDT DNA) was performed in 50 mM Hepes pH 7.5, 100 mM NaCl, and 1 mM MgCl_2_. Complementary single strand DNA were annealed and purified on a SOURCE 15Q anion exchange column (GE Life Sciences). Methylated DNA was ordered with an internal CpG symmetrically methylated once annealed (5′-FAM-CTGCCGC(mC)GAGCGCCTC-3′). A DNA substrate devoid of CpG sites (CpG-free) was identically annealed and purified (5′-FAM-CTGGCCCCAGGGCCCTC-3′). Protein was serially diluted with DNA (10 nM) before measuring polarization on a CLARIOstar microplate reader (BMG Labtech). Binding isotherms were fit in DataGraph (Version 4.6, Visual Data Tools, Inc, Chapel Hill, NC; https://www.visualdatatools.com/) by a standard sigmoidal equation as previously described (25).

### Surface plasmon resonance

Binding affinities of MBD2sc and MBD3sc to biotinylated DNA matching our FP substrates was performed on a Biacore 8K (Cytiva). We used a buffer of 50 mM Hepes pH 7.5, 150 mM NaCl, 1 mM MgCl2, 1 mM TCEP, and 0.05% TWEEN-20 flowing at 30 μl/min. DNA was immobilized to a SA sensor chip by injecting 100 nM of each substrate for 60 s resulting in a response of ∼350. Protein was rapidly injected at consecutively increasing concentrations, single-cycle kinetics (56), each lasting 120 s and a final dissociation of 600 s. Response curves were fit using the Biacore Insight Evaluation software (Fig. S9).

### DNA substrates for single-molecule imaging

To generate a DNA substrate incorporating a known target sequence for MBD2, we subcloned a portion (3837 bps) of the death-associated protein kinase 1 (DAPK1) promoter (chromosome 9, bases 87497573–87501409), which includes CpG-rich (4689 bps) and CpG-poor (2150 bps) regions. This sequence was then subdivided into clones that contain only the CpG-rich region (CpG-rich: 4705 bps) or CpG-rich plus poor regions (CpGrich-poor: 6839 bps) within the pGEM backbone. For a CpG-free DNA substrate, we purchased the pCpGfree-vitroNmcs plasmid (5488 bps) from InvivoGen, which does not contain any CpG dinucleotides. The CpG-rich region from the DAPK1 promoter was then subcloned into the pCpGfree-vitroNmcs plasmid at the ScaI/NcoI restriction sites to generate a 1697 bps CpG-rich region within a 5466 bps CpG-free backbone (CpG-free–rich DNA: 7163 bps). In addition, we subcloned a much smaller CpG rich region derived from the DAPK1 CpG-rich sequence into the Sca/NcoI restriction sites in the pCpGfree-vitroNmcs plasmid (CpG-mini: 140 bps). Notably, the StuI restriction site in pCpGfree-vitroNmcs is equidistant from both ends of the cloned fragment such that StuI digestion generates a linear fragment with the various inserts located in the middle.

We treated purified plasmids with CpG methyltransferase (M.SssI) and SAM cofactor at 37 °C for 2 h to generate methylated DNA substrates. Restriction digest with HpaII confirmed complete DNA methylation (Fig. S2). A total of 30 HpaII sites are present in the CpG-rich substrate. For DNA tightropes, we linearized DNA substrates with StuI (New England BioLabs) digestion and ligated the DNA using the Quick Ligation Kit (New England BioLabs) at room temperature overnight. Finally, we purified the ligated DNA samples by phenol-chloroform extraction.

### Protein-QD conjugation

We purchased SAv-QDs (SAv-QDs-655) from Invitrogen. For QD labeling of N-terminal His_6_-tagged MBD2sc and MBD3sc, we incubated 0.7 μl of SAv-QDs-655 (1 μM) with 1.5 μl of the multivalent chelator tris-nitrilotriacetic acid (2 μM) for 10 min at room temperature (39). We then added the His-MBD proteins (0.5 μl of MBD2 and 1.0 μl of MBD3, each 4 μM) to the SAv-QD-nitrilotriacetic acid solution and incubated for an additional 10 min at room temperature. Finally, we diluted all samples 100× before injecting them into the flow cell in the imaging buffer (50 mM Hepes pH 7.5, 100 mM NaCl, 1 mM MgCl_2_, and 1 mg/ml bovine serum albumin).

### Fluorescence imaging and analysis

The oblique angle total internal reflection microscopy–based particle tracking of QD-labeled proteins on DNA tightropes was performed as described previously (37). Briefly, we collected images with an inverted microscope (Nikon Ti-E) with a 100 × objective (APO TIRF, Nikon). Red (655 nm) QD-protein complexes were excited at 488 nm by a solid-state laser (Sapphire DPSS). The signal was split into two channels using a dichroic mirror (T605LPXR, Chroma) and passed through an emission filter (ET655/40 nm, Chroma). We assembled flow cells as described in previous studies (37). We immobilized poly-L-lysine (2.5 mg/ml, MW > 300 KDa, Wako Chemicals)–treated silica beads onto a coverslip surface with PEGylation and then introduced ligated DNA substrates into the flow cell with a syringe pump at a flow rate of 300 μl/min. DNA tightrope length was controlled by adjusting the silica bead coverage resulting in an average length of 12.5 μm (Fig. S4*B*). The quality of the DNA tightropes is determined after each experiment by adding YOYO-1 Iodide dye (Thermo Fisher) to the flow cell at a concentration of 0.4 μM. Any datasets with an observable relaxed or flexible tightrope appearance are discarded.

All images were collected using an EMCCD (iXon DU897, Andor Technology) at a 50 ms/frame time resolution.

The MSD as a function of time is given by:

(1) $$MSD\left(n\Delta t\right)=\frac{1}{N-n}\sum_{i=1}^{N-n}\left[{\left(x_{i+n}-x_{i}\right)}^{2}\right]$$

where N is the total number of frames in the trajectory, n is the number of frames for different time intervals, Δt is the time between frames, and *x*_*i*_ is the position of the protein-QD in the frame *i.* We determined the 1-D diffusion coefficient (*D*) and alpha exponent (α) by a custom routine developed in LabView Software based on the following equation:

(2) $$MSD=2Dt^{\alpha }$$

We categorized a protein as mobile if the diffusion coefficient was greater than 1X10^-5^ μm^2^/s and the R^2^ value from data fitting (Equation 2) exceeded 0.9. We analyzed the diffusion range using a custom MATLAB script.

### AFM imaging and image analysis

All DNA and protein samples were preincubated for 20 min at room temperature, diluted 10× in AFM buffer (25 mM NaOAc, 25 mM Hepes–KOH (pH 7.5), and 10 mM Mg(OAc)_2_), and deposited onto a freshly cleaved mica surface (SPI Supply). The samples were washed with purified water (MilliQ) and dried with nitrogen gas. The final concentration of substrates deposited onto mica was ∼0.5 ng/μl and 30 nM for DNA and protein, respectively. All images were obtained using the tapping-mode in air on an MFP-3D-Bio AFM (Asylum Research). We used cantilevers (PPP-FMR, Nanosensors) with spring constants at ∼2.8 N/m and collected images at a scan size of 3 μm x 3 μm, a scan rate of 1 to 2 Hz, and a resolution of 512 × 512 pixels. The DNA-bending angle was analyzed using Asylum software.

### Statistical analysis

The statistical significance level based on one-way ANOVA with Tukey’s test for *post-hoc* analysis was set at *p* < 0.05 (SPSS version 27, IBM).

## Data availability

All data presented is contained within this article and is available from the authors upon request.

## Supporting information

This article contains supporting information.

## Conflict of interest

The authors have no conflicts of interest to declare regarding the publication of this article.
