# Supplementary material for: Densely methylated DNA traps Methyl-CpG–binding domain protein 2 but permits free diffusion by Methyl-CpG–binding domain protein 3
Source: J Biol Chem. 2022 Aug 28;298(10):102428. doi: 10.1016/j.jbc.2022.102428 (PMC9520026; doi:10.1016/j.jbc.2022.102428)
Supplement: Supplementary [file mmc1.pdf]

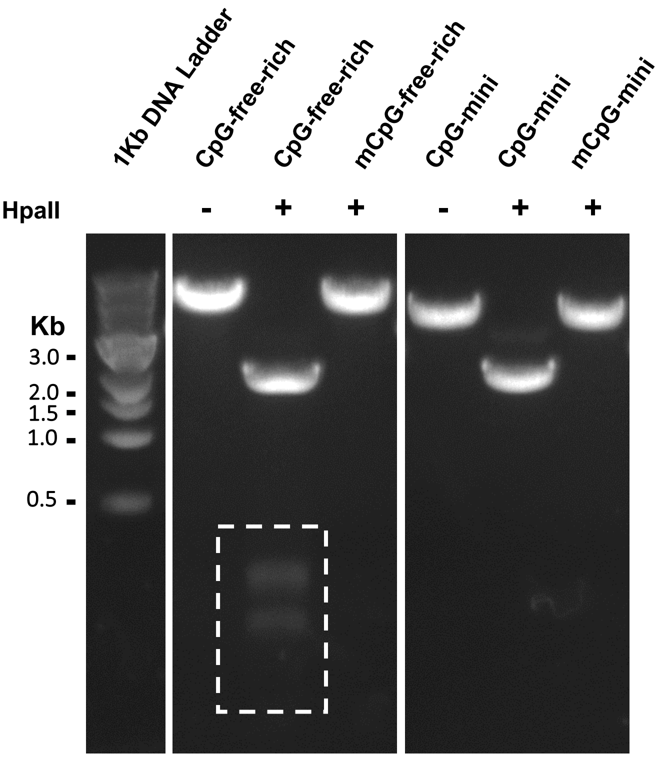

Figure S1. Confirmation of DNA methylation. DNA substrates were digested with the methylation-sensitive HpaII restriction enzyme**.** The boxed regions indicate digested DNA fragments from unmethylated DNA. All lanes were compiled from images of the same agarose gel stained with SYBR Safe (Thermo Fisher Scientific).

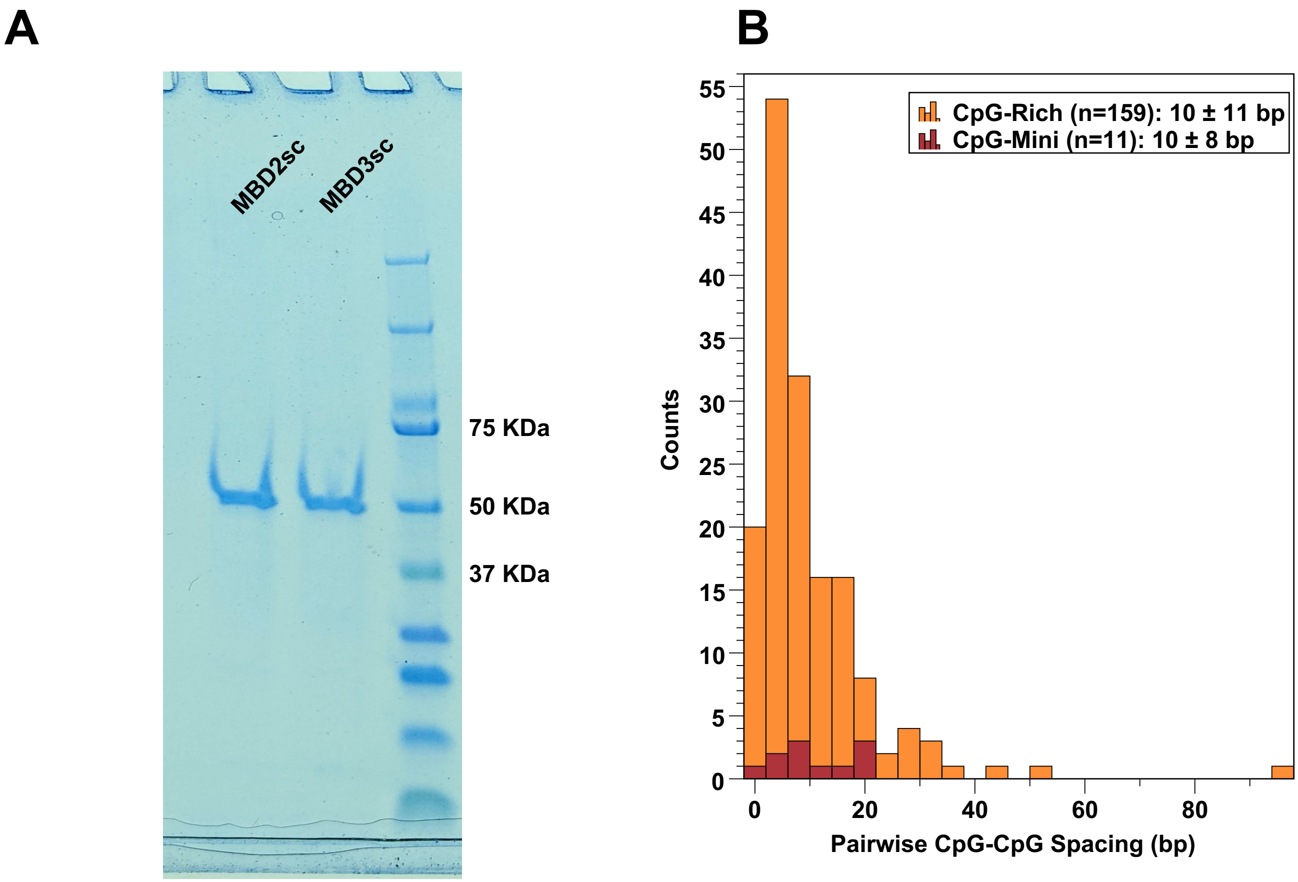

Figure S2. (**A**) SDS-PAGE of purified MBD2sc and MBD3sc proteins (~51 KDa). (**B**) Histogram of pairwise CpG-CpG spacing comparing the CpG-rich and CpG-mini DNA substrates.

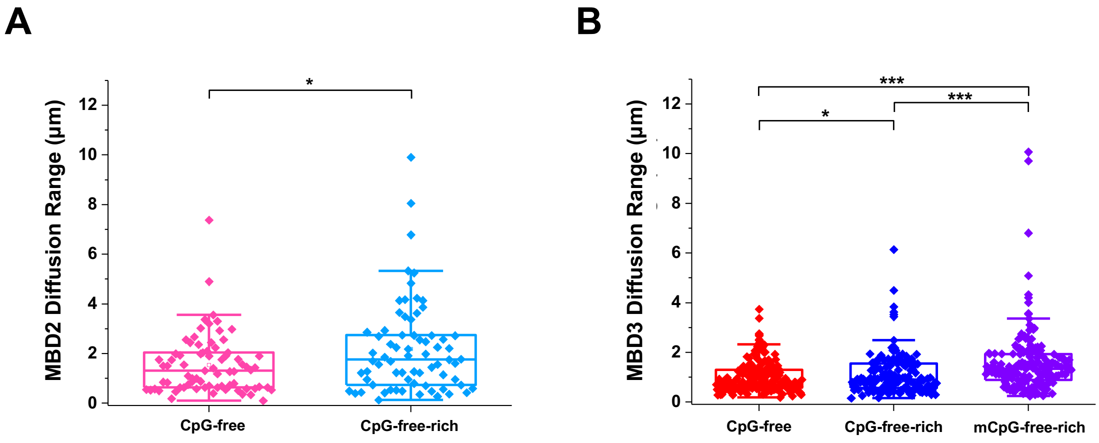

Figure S3. Diffusion ranges of MBD2sc and MBD3sc on different DNA substrates.
(**A**) The diffusion ranges of MBD2sc on CpG-free DNA tightropes is 1.5 (± 1.0) μm (N = 99), and 2.2 (± 1.4) μm on CpG-free-rich tightropes (N = 100). (**B**)The diffusion ranges of MBD3sc is 1.0 (± 0.6) μm on CpG-free DNA tightropes (N = 199), 1.1 (± 0.8) μm on CpG-free-rich tightropes (N = 144), and 1.6 (± 1.2) μm on mCpG-free-rich tightropes (N = 174). *: $p < 0.05$; ***: $p < 0.001$.

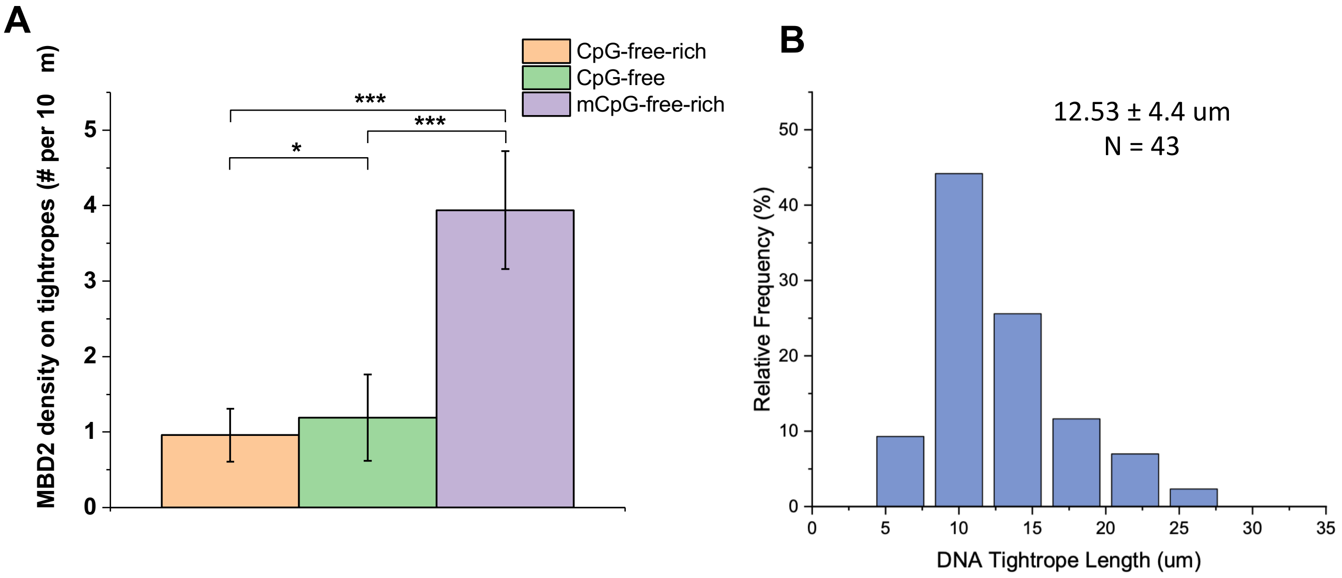

Figure S4. Quantification of the MBD2sc-QD density on different DNA tightropes. **(A)** Numbers of MBD2sc-QD per 10 μm of DNA tightrope length: 0.96 ± 0.35 for CpG-free-rich (N = 124), 1.19 ± 0.57 for CpG-free (N = 137), and 3.97 ± 0.78 for mCpG-free-rich (N = 352). *: $p < 0.05$; ***: $p < 0.001$. (**B**) A histogram of the length of DNA tightropes formed from a subset of the various DNA substrates. On average the bead spacing is 12.53 +/- 4.4 μM consisting of 5-6 linearly ligated plasmids.

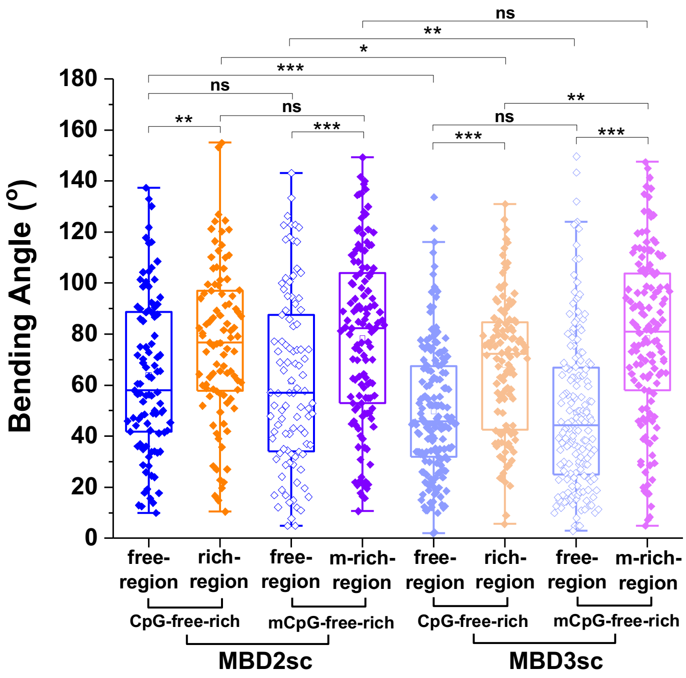

Figure S5. Comparison of the DNA bending angles induced by MBD2sc and MBD3sc. Box plots showing the DNA bending angles induced by binding of MBD2sc and MBD3sc to CpG-free, and unmethylated and methylated CpG-rich regions on the linear unmethylated and methylated CpG-free-rich DNA substrates. *: $p < 0.05$; **: $p < 0.005$; ***: $p < 0.001$.

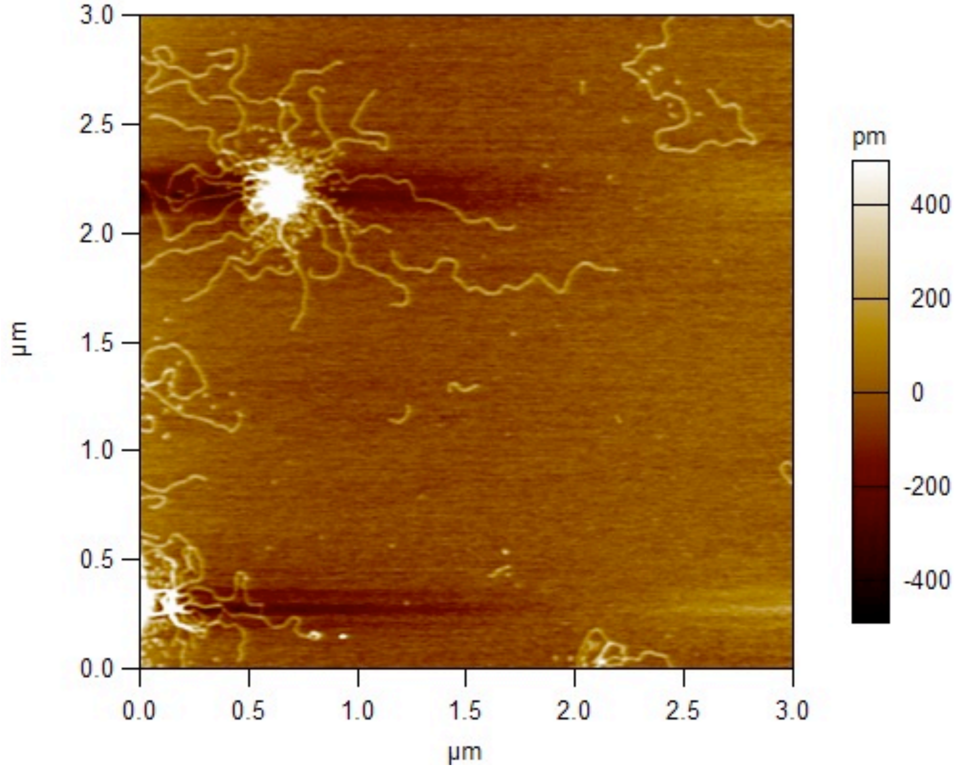

Figure S6. MBD2sc:DNA cluster formation. AFM image of mCpG-free-rich DNA incubated with a high concentration of MBD2sc shows formation of large clusters. [MBD2sc] = 1.2 µM. [mCpG-free-rich] = 0.5 ng/µL.

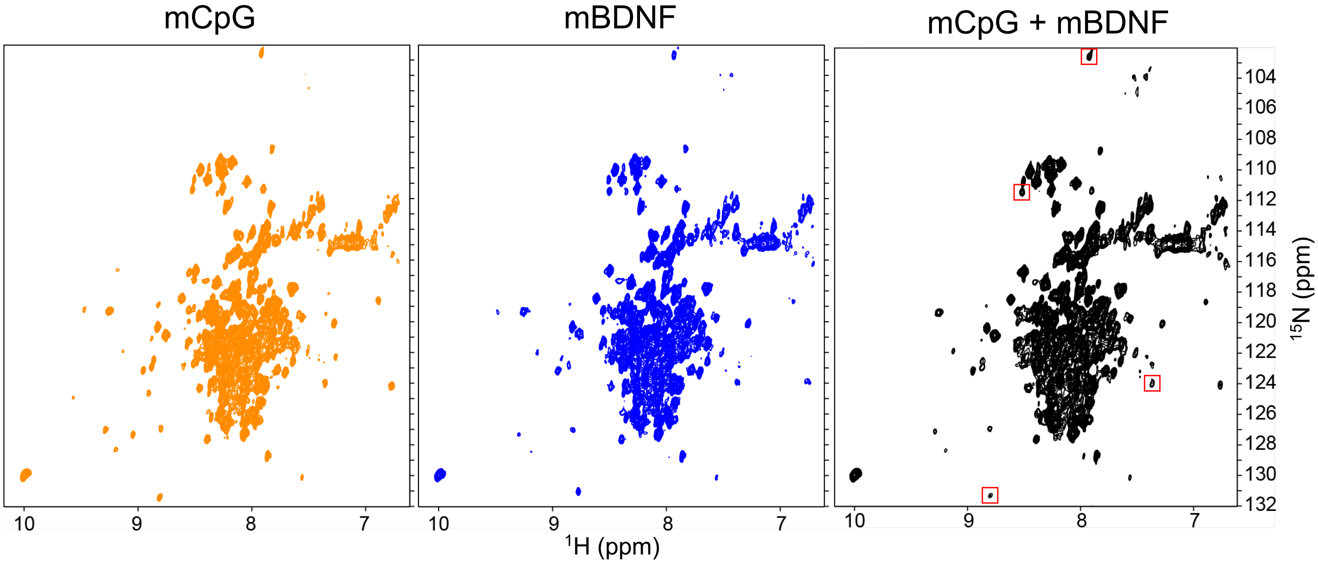

Figure S7. NMR spectra of MBD2sc on methylated DNA. 2D 1H-15N TROSY-HSQC of MBD2sc on three differently methylated DNA substrates; mCpG, mBDNF, or both (mCpG + mBDNF). Resonances corresponding to the DNA binding domain exhibit unique chemical shifts depending on which methylated site MBD2sc is bound. Highlighted residues in red are not significantly broadened when both DNA sites are methylated and are overlayed in Figure 8.

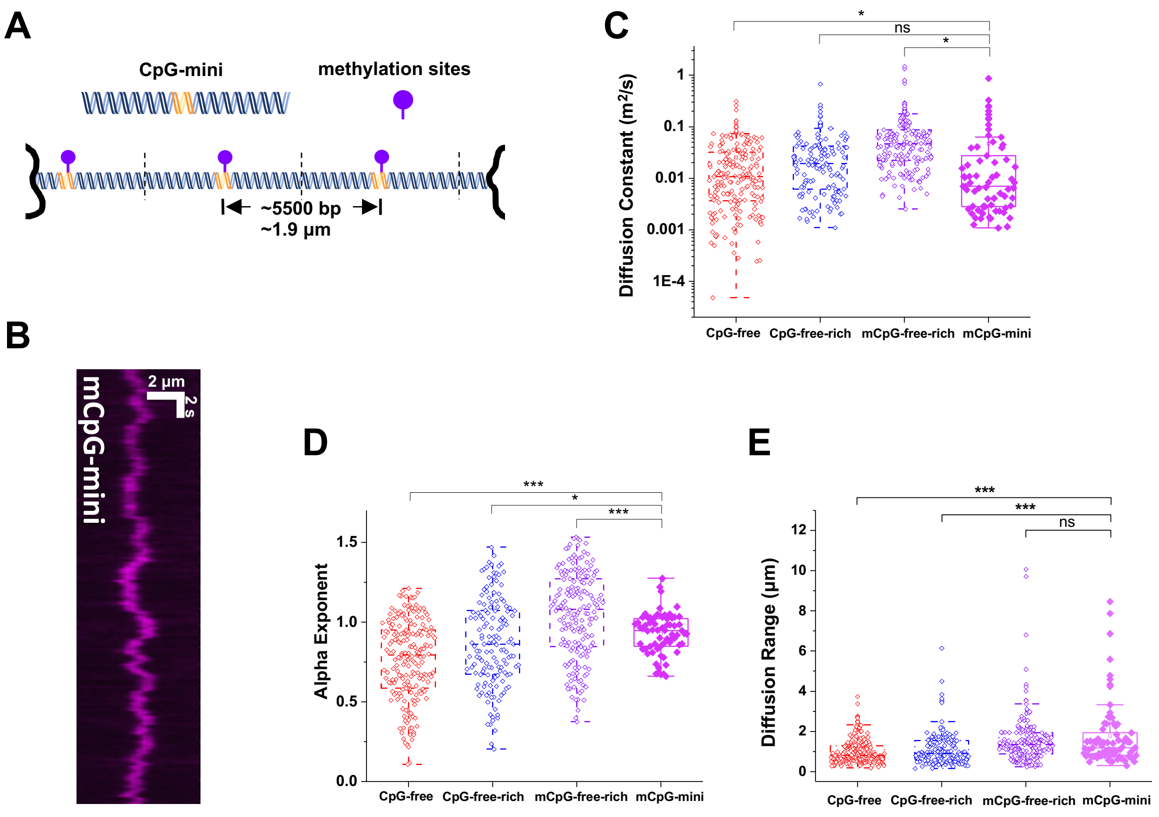

Figure S8. MBD3sc conducted 1-D free diffusion on mCpG-mini DNA tightropes. (**A**) A Cartoon drawing of the ligated mCpG-mini DNA substrates for the DNA tightrope assay. (**B**) A representative kymograph of MBD3sc diffuses on the ligated mCpG-mini DNA tightrope. The diffusion constant (**C**), the alpha exponent (**D**), and the diffusion range (**E**) of MBD3sc on mCpG-mini. For direct comparison, the data for MBD3sc binding to the other DNA substrates is re-plotted from **Figures 5 and S3B** (shown as open symbols and dashed lines). *: $p < 0.05$; **: $p < 0.005$; ***: $p < 0.001$.

	CpG Free Rich				mCpG Free Rich				CpG free				mCpG-mini			
	MBD3 (n=123)		MBD2 (n=147)		MBD3 (n=345)		MBD2 (n=246)		MBD3 (n=240)		MBD2 (n=333)		MBD3 (n=158)		MBD2 (n=253)	
	Avg	SD	Avg	SD	Avg	SD	Avg	SD	Avg	SD	Avg	SD	Avg	SD	Avg	SD
**Cluster**	15	8	2	2	2	1	2	2	6	1	0	0	3	2	2	2
**Single**	85	8	98	2	98	1	98	2	94	1	100	0	97	2	98	2

Table S1. Percentage of clustered/single proteins on DNA tightropes.

The percentage of single protein or cluster of QD observed on DNA tightrope substrates based on QD blinking. These data show that the majority of all imaged protein-QD are single proteins.

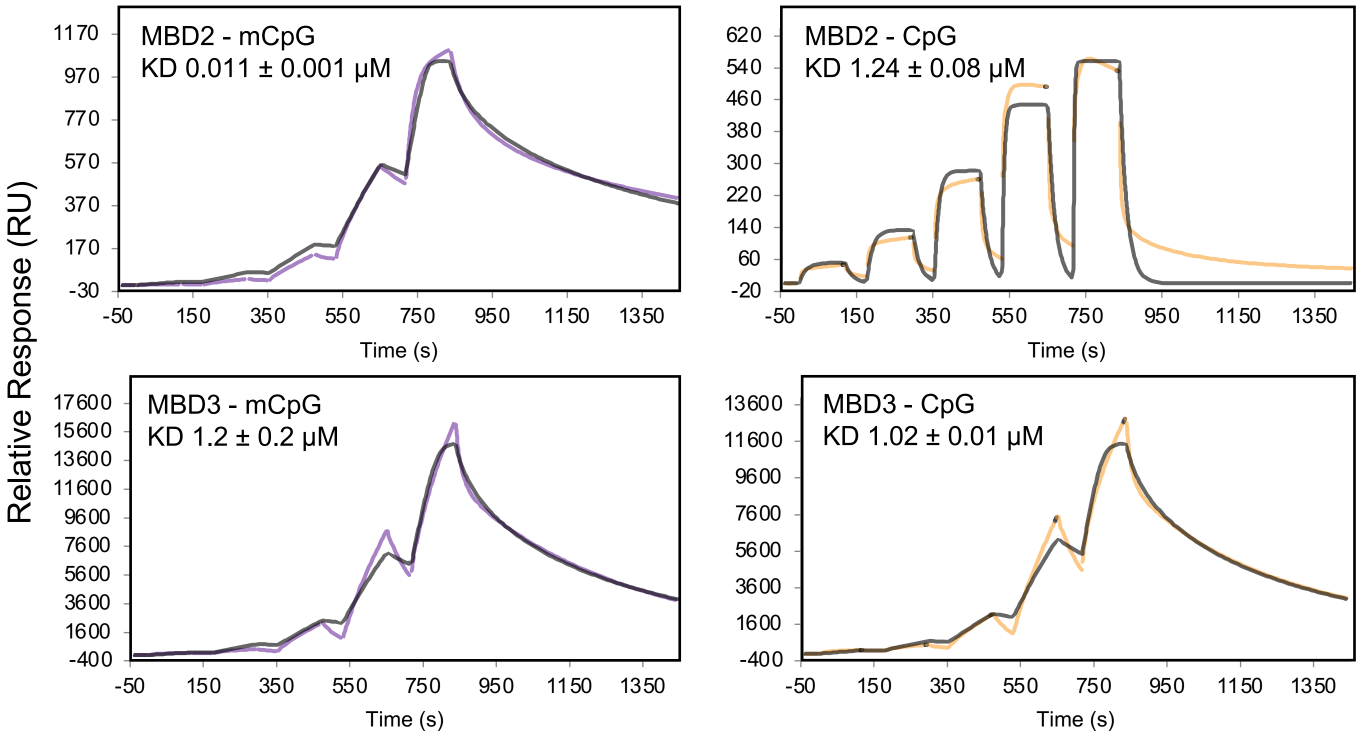

Figure S9. MBD2/3sc binding affinity to CpG substrates by SPR.
The binding affinity of MBD2sc (top row) and MBD3sc to methylated (purple) or unmethylated (orange) DNA as measured by SPR. Increasing concentrations of protein were injected for 120 seconds each using single-cycle kinetics. Protein concentrations are highest at 0.15 or 9 µM, depending on the system, and diluted 3-fold. Response curves are fit using the Biacore Insight Evaluation software (black lines).
